# A Cell Wall Hydrolase MepH Is Negatively Regulated by Proteolysis Involving Prc and NlpI in *Escherichia coli*

**DOI:** 10.3389/fmicb.2022.878049

**Published:** 2022-03-28

**Authors:** Wook-Jong Jeon, Hongbaek Cho

**Affiliations:** Department of Biological Sciences, College of Natural Sciences, Sungkyunkwan University, Suwon, South Korea

**Keywords:** gram-negative bacteria, cell wall, DD-endopeptidase, peptidoglycan crosslinks, periplasmic protease, proteolytic control

## Abstract

Cell wall assembly of Gram-negative bacteria requires DD-endopeptidase activity that cleaves peptidoglycan (PG) crosslinks in addition to PG synthetic activity, and the activity of DD-endopeptidases needs to be tightly regulated to maintain cell wall integrity during PG expansion. Among the major DD-endopeptidases functioning for PG assembly in *Escherichia coli*, MepS and MepM have been shown to be negatively controlled by the periplasmic protease Prc. In this study, we performed a genetic selection using the synthetic lethality between the *mepS* and *mepM* mutations in rich medium to uncover regulatory mechanisms controlling the activity of DD-endopeptidases other than MepS and MepM. This selection revealed mutations in *prc* and *nlpI* as suppressors. Gene deletion analyses revealed that MepH is required for suppression of the MepS^—^ MepM^—^ growth defect by the *prc* or *nlpI* mutation. We also discovered that MepH is directly degraded by Prc and that this degradation is further promoted by NlpI. Thus, our study showed that all three DD-endopeptidases which play major roles in PG assembly of *E. coli* under normal physiological conditions are controlled by a common periplasmic protease.

## Introduction

Peptidoglycan (PG) is a giant mesh-like molecule that surrounds bacterial cells outside the cytoplasmic membrane, functioning as a cell wall that protects bacteria from osmotic rupture. PG consists of glycan strands crosslinked by short peptides attached to the glycan strands. Thus, PG synthesis requires glycosyltransferase activity to polymerize glycans and transpeptidase activity to crosslink the glycan strands. In addition to the synthesis activity, PG assembly also requires endopeptidase activity to cleave peptide crosslinks, because the PG network needs to be opened to insert newly synthesized PG (Hashimoto et al., 2012; Singh et al., 2012; Vollmer, 2012). In Gram-negative bacteria, DD-endopeptidase activity that cleaves the 4–3 peptide crosslinks of PG has been shown to be essential for PG assembly and survival (Singh et al., 2012; Dörr et al., 2013; Srivastava et al., 2018; Murphy et al., 2021).

In *Escherichia coli*, there are eight periplasmic enzymes that exhibit DD-endopeptidase activity. Among these, MepS and MepM are thought to play a major role in cell wall expansion because inactivation of both enzymes causes defective PG assembly and cell lysis in rich medium (Singh et al., 2012). MepH is thought to play an important role in PG assembly in the absence of MepS and MepM because it is required for survival of the *mepS mepM* mutant in minimal medium (Singh et al., 2012). Among the remaining DD-endopeptidases, MepK exhibited weak DD-endopeptidase activity *in vitro*, but is thought to function mainly as an LD-endopeptidase that cleaves 3–3 peptide crosslinks (Chodisetti and Reddy, 2019). The cellular functions of the remaining DD-endopeptidases, PBP4 (*dacB*), PBP7 (*pbpG*), MepA, and AmpH, in cell wall assembly has remained largely unclear.

The activity of PG hydrolases needs to be tightly regulated in time and space to prevent lethal damage to the cell wall. In *E. coli*, it was previously shown that MepS levels are negatively controlled by the periplasmic protease Prc and the lipoprotein NlpI that functions as an adaptor between Prc and MepS (Singh et al., 2015; Su et al., 2017). Recently, MepM was reported to be another substrate of Prc, but unlike MepS, proteolytic control of MepM did not depend on NlpI (Kim et al., 2021). Similar negative regulation of MepS and MepM homologs by a proteolytic system consisting of the periplasmic protease CtpA and the lipoprotein adaptor LbcA was discovered in *Pseudomonas aeruginosa*, suggesting that proteolytic control of DD-endopeptidase activity is widely conserved among Gram-negative bacteria (Srivastava et al., 2018).

In addition to proteolytic control, transcriptional regulation of DD-endopeptidase expression has also been reported. *Vibrio cholerae* has three *mepM* homologues, *shyA*, *shyB*, and *shyC*, among which *shyA* and *shyC* are collectively required for growth in LB (Dörr et al., 2013). The *shyA shyC* double mutant was recently reported to be able to survive in minimal medium because *shyB* expression is induced by the transcriptional activator Zur in zinc-depleted minimal medium (Murphy et al., 2019). Although all three enzymes show homology to a zinc-dependent metallopeptidase, ShyB either has a much higher affinity to zinc than ShyA or ShyC, or functions without zinc. The transcriptional regulation of *shyB* expression is thought to ensure PG assembly during zinc starvation.

The goal of this study was to investigate the mechanisms that control the level or activity of DD-endopeptidases other than MepS and MepM in *E. coli*. We expected that revealing the regulatory mechanisms might provide clues to understand the cellular functions of these DD-endopeptidases. To identify regulatory factors, we performed a genetic selection screen for mutations that suppress the synthetic lethality between *mepS* and *mepM*, which uncovered the *prc* and *nlpI* mutations as suppressors. Further analyses of these mutations revealed that the proteolytic system consisting of Prc and NlpI is involved in the negative regulation of MepH.

## Materials and Methods

### Bacterial Strains and Growth Conditions

Strains and plasmids used in this study are listed in Supplementary Tables S1 and S2. All *E.coli* strains used in the reported experiments are derivatives of MG1655 (Guyer et al., 1981). Bacterial cells were grown in lysogeny broth (LB; 1% tryptone, 0.5% yeast extract, 0.5% NaCl) or minimal M9 medium supplemented with a carbon source (0.2% glucose or arabinose). Antibiotics were used at 25 μg/ml (chloramphenicol; Cm), 37.5 μg/ml (kanamycin; Kan), or 50 μg/ml (ampicillin; Amp).

### Strain Construction *via* Recombineering

#### Gene Deletion

Gene deletions were constructed to resemble those in the Keio knockout collection (Baba et al., 2006). The Kan^R^ cassette was amplified by using pKD13 as a template with the primer pairs listed in Supplementary Table S3. Sequences homologous to the chromosomal sequence are underlined. The amplified DNA was electroporated into TB10 that expresses the lambda Red genes and recombinants were selected on LB agar supplemented with kanamycin (Johnson et al., 2004).

#### Replacement of the Native *mepS* Promoter With an Arabinose-Inducible Promoter

The native promoter of the *mepS* gene was replaced with an arabinose-inducible promoter, as previously described (Bernhardt and Boer, 2003). The (*aph araC* P_ara_) cassette from pTB29 was amplified with the primer pair listed in Supplementary Table S3. The resulting DNA fragment was flanked on either side by 40 bp sequences homologous to the region upstream of *mepS* (underlined in the primer sequences). This DNA was electroporated into TB10 and recombinants were selected on LB agar supplemented with kanamycin.

#### Construction of 3X-FLAG-Tagged Genes at the Native Chromosomal Loci

The 3X FLAG epitope tagging at the 3′ end of a gene at its native chromosomal locus was also performed by recombineering using TB10. The 3X-FLAG Kan^R^ marker cassette from pHC965 was amplified with the primer pairs listed in Supplementary Table S3. The resulting DNA fragment was flanked by on either side by 40 bp sequences homologous to the region at the 3′ end of each target gene. This DNA was electroporated into TB10 and recombinants were selected on LB agar supplemented with kanamycin to add the 3X-FLAG Kan^R^ sequence to the 3′ end of each gene.

### Construction of Strains With Multiple Deletions

Strains with multiple deletion mutations were made by sequential introduction of each deletion *via* P1 transduction followed by removal of the *aph* cassette using FLP recombinase expressed from pCP20, leaving a *frt* scar sequence at each deletion locus (Datsenko and Wanner, 2000). The correct orientation of the DNA flanking *frt* sequences in multiple deletion mutants was confirmed for all deletions in each mutant.

### Microscopy

Growth conditions prior to microscopy are described in the figure legends. Prior to imaging, cells were immobilized on 2% agarose pads containing 1X M9 salts and covered with #1.5 coverslips. Micrographs were obtained using a Leica DM2500 LED microscope equipped with a Leica DFC7000 GT camera, Fluo Illuminator LRF 4/22, HC PL APO 100x/1.40 Oil Ph3 objective lens, and Leica Las X acquisition software.

### Generation of the Transposon Insertion Library

HC611 was mutagenized with the Kan^R^ mariner transposon delivered by conjugation from the donor strain MFD*pir*/pSC189, which is auxotrophic for diaminopimelic acid (DAP; Ferrières et al., 2010). The donor strain was grown in LB supplemented with 300 μM DAP and 50 μg/ml ampicillin. The recipient strain HC611 was grown in M9-arabinose medium supplemented with 0.2% casamino acids. The cells were washed and resuspended in LB supplemented with 0.2% arabinose and 300 μM DAP to 1/10th of the original culture volume. Equal volumes of donor and recipient were mixed together and 50 μl aliquots were spotted on LB agar supplemented with 0.2% arabinose and 300 μM DAP. Donor-only and recipient-only aliquots were also spotted in parallel to verify proper selection of transconjugants. Mating was allowed to proceed at 37°C for 3 h. Cells were resuspended in LB and transconjugants were then selected on LB agar supplemented with 0.2% arabinose and 37.5 μg/ml kanamycin overnight at 37°C. About 1.4 × 10^5^ colony-forming units were scraped from agar plates by suspension in LB, glycerol was added to a final concentration of 15%, and aliquots were frozen at −80°C.

### Suppressor Selection

An aliquot of the transposon mutant library of HC611 was thawed and serially diluted in LB. The serial dilutions were spread on LB agar and incubated at 37°C to select for suppressors of the *mepM mepS* synthetic lethality. The serial dilutions were also spread on LB agar supplemented with 0.2% arabinose to determine the suppressor frequency. The suppressor frequency of the transposon mutant library was 1.6 × 10^−3^, which was about 20-fold higher than the mock library of HC611. The transposon insertion sites of the suppressors were determined by arbitrarily primed PCR (ArbPCR). The first round of ArbPCR was performed with the primer pair 5′- GGCCACGCGTCGACTAGTACNNNNNNNNNNGATGC - 3′ and 5′- ATCGGCAAAATCCCTTATAAATC -3′. The second nested PCR was performed with 5′- GGCCACGCGTCGACTAGTAC -3′ and 5′- AATAGGAACTTCGGAATAGGAAC -3′ to increase specificity and sensitivity. The resulting PCR products were sequenced with the primer 5′- AATAGGAACTTCGGAATAGGAAC -3′ that anneals to the transposon sequence to identify the transposon–chromosome junctions.

### Immunoblotting

Cells in 2 ml cultures with an OD_600_ of about 0.5 were harvested by centrifugation. Cell pellets were resuspended in 0.1 ml 1X Laemmli sample solution, sonicated, and heated at 95°C for 5 min. Then, proteins were separated by SDS/PAGE and transferred onto a PVDF membrane. To reduce non-specific binding, the membrane was blocked by incubation in 5% skimmed milk in 1X PBS containing 0.05% Tween-20 (PBS-T) for 2 h. The blocked membrane was then incubated overnight with the appropriate primary antibodies, monoclonal anti-FLAG M2 antibody (Sigma, 1:10,000) or anti-RpoA 4RA2 Mouse IgG1 (BioLegend, 1:4,000), at 4°C. The membrane was washed four times with PBS-T and then probed with appropriate secondary antibodies conjugated with horseradish peroxidase, anti-mouse IgG HRP-linked antibody (Cell Signaling Technology, 1:20,000), and incubated for 3 h at room temperature. After washing the membrane with 1× PBS-T five times, the membrane was overlaid with ECL-SuperKine^™^ West Femto Maximum Sensitivity Substrate (Abbkine) for anti-FLAG immunoblotting or Miracle-Star^™^ Western blot detection system (iNtRON) for anti-RpoA immunoblotting, and chemiluminescence was imaged using the ImageQuant LAS 4000 mini imaging system (GE healthcare).

### Protein Purification

Prc, NlpI, and MepH were overexpressed and purified with a 6xHis-SUMO (H-SUMO) tag fused to the N-terminus (Mossessova and Lima, 2000; Marblestone et al., 2006). Before the protein purification steps, the plasmid clones, pWJ24, pWJ25, or pWJ26, were transformed into the *E.coli* BL21(DE3)/pLysS strain and the fresh transformants were inoculated into 10 ml of LB supplemented with ampicillin, chloramphenicol, and 0.2% glucose and grown overnight at 37°C. These cultures were diluted 1:100 into 0.5 liter of LB containing ampicillin and 0.04% glucose, and grown at 37°C. When the OD_600_ reached 0.5, protein expression was induced by the addition of 1 mM IPTG and grown at 30°C for an additional 4 h. Then, cells were centrifuged at 6,000 × g for 10 min at 4°C. The cell pellets were resuspended in 25 ml of 1X PBS (Bio-Rad, 10 mM sodium phosphate, 150 mM sodium chloride, pH 7.8) supplemented with 20 mM imidazole, and stored at −80°C. The resuspended cell pellets were lysed by sonication and centrifuged at 50,000 × g for 90 min at 4°C to remove cell debris. The supernatant was loaded onto 1 ml of Ni^2+^-NTA agarose equilibrated with 1X PBS containing 20 mM imidazole. The column was then washed with 20 ml of 1X PBS containing 20 mM imidazole. Bound protein was eluted sequentially with 1 ml of 1X PBS containing increasing amounts of imidazole (50, 100, 150, 200, 250, 300, or 500 mM). The peak fractions containing the eluted proteins were pooled, mixed with 50 μg His6-tagged UlpI (SUMO protease) to cleave the His6-SUMO-tag, and dialyzed against 1X PBS overnight at 4°C to remove imidazole. The dialyzed protein was passed onto 1 ml of Ni^2+^-NTA agarose equilibrated with 1X PBS to remove the released His6-SUMO tag, His6-tagged UlpI, and other contaminating proteins. Untagged Prc and NlpI were collected in the flow through, aliquoted, snap-frozen in liquid nitrogen, and stored at −80°C.

MepH was soluble in the His-SUMO-tagged form but precipitated after cleavage of the His-SUMO tag. Thus, MepH was prepared in the form of His-SUMO-MepH by dialyzing the peak fractions of His-SUMO-tagged MepH in 1X PBS without treatment of UlpI.

## Results

### Overexpression of DD-Endopeptidases Can Suppress the Synthetic Lethality Between *mepS* and *mepM* Mutations

**Figure 1:**
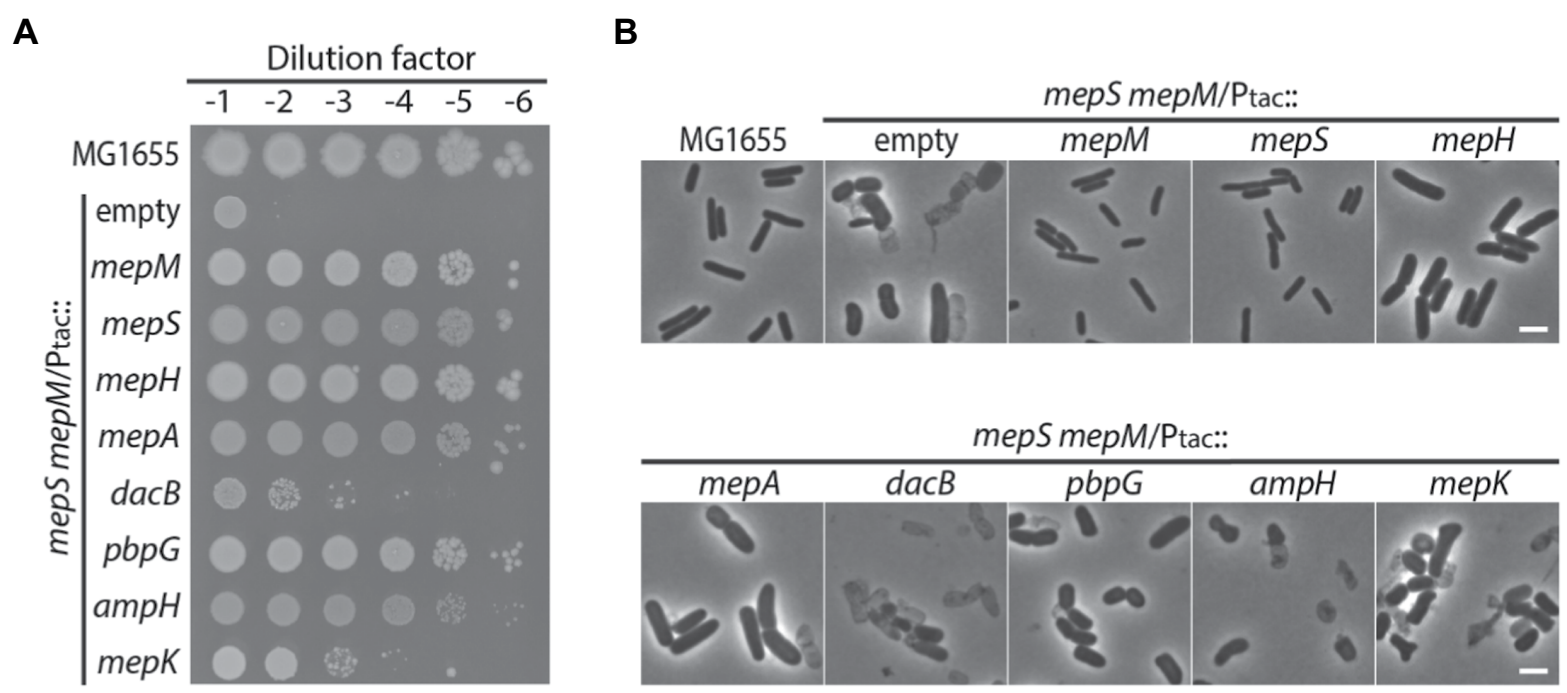
DD-endopeptidases other than MepS, MepM, and MepH can also function in cell wall assembly. **(A)** Spot dilution assay on LB agar for MG1655 (wild type) and HC611 (P_ara_::*mepS*, *ΔmepM*) strains harboring plasmids that overexpress various DD-endopeptidase genes: pHC800 (empty vector), pTK1(*mepM*), pTK2(*mepS*), pTK3(*mepH*), pTK4(*mepA*), pTKD1(*dacB*), pTKD4(*pbpG*), pTKD5(*ampH*), or pWJ20(*mepK*). The strains were grown overnight in M9-arabinose medium and normalized for cell density to an OD_600_ of 2. The normalized cultures were serially diluted 10-fold in LB and 5 μl of each dilution (10^−1^–10^−6^) was spotted onto LB agar lacking arabinose but containing 1 mM IPTG to deplete *mepS* expression and induce the expression of various DD-endopeptidases from the *tac* promoter. The plates were incubated at 30°C and photographed after 22 h. **(B)** Phase contrast images of the same strains after growth in LB. Overnight cultures were washed with LB and diluted to an OD_600_ of 0.01 in LB containing 1 mM IPTG. Growth was then continued at 37°C with agitation. After incubation for 4 h, samples of the cells were chemically fixed and the cells were imaged using phase contrast optics. The bars equal 3 μM.

Among the genes that encode DD-endopeptidases in *E. coli*, *mepS*, *mepM*, and *mepH* have been shown to be collectively required for cell wall assembly, but the cellular functions of the other DD-endopeptidases remain unclear. To examine whether other DD-endopeptidases can also function in PG assembly *in vivo*, we tested if overexpression of individual DD-endopeptidase genes suppresses the growth defect of the *mepS mepM* double mutant in LB medium. To facilitate characterization of the MepS^—^ MepM^—^ growth defect, we constructed the strain HC611 (P_ara_::*mepS*, *ΔmepM*) whose *mepS* promoter was replaced with the *araBAD* promoter in combination with the *mepM* deletion. Then, plasmids that express each DD-endopeptidase gene from the IPTG-inducible *tac* promoter were introduced into HC611. Suppression of the MepS^—^ MepM^—^ growth defect was tested on LB agar lacking arabinose but instead supplemented with IPTG to deplete *mepS* expression and induce the expression of the DD-endopeptidases. In addition to *mepS* and *mepM*, overexpression of *mepH*, *pbpG*, *mepA*, and *ampH* also suppressed the growth defect (Figure 1A). Although strains overexpressing DD-endopeptidases other than *mepS* and *mepM* formed much wider cells than the wild-type strain, overexpression of *mepH*, *pbpG*, and *mepA* prevented the lysis of HC611 in LB medium, showing that these DD-endopeptidases can at least function in maintaining cell wall integrity (Figure 1B).

### Inactivation of *prc* or *nlpI* Suppresses the Synthetic Lethality of the *mepS* *mepM* Mutant

**Figure 2:**
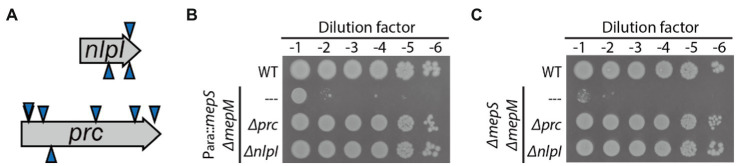
Inactivation of *prc* or *nlpI* suppresses the synthetic lethality between *mepS* and *mepM* mutations. **(A)** A diagram of the transposon insertion sites of suppressors isolated from the selection of a random transposon insertion library of HC611 (P_ara_::*mepS*, *ΔmepM*) on LB agar. The triangles above the gene arrow represent the insertion for which the direction of transcription in the kanamycin resistance cassette of the transposon is in the same orientation as the disrupted gene; the triangles below, the opposite direction. **(B)** MG1655, HC611, and the *Δprc* and *ΔnlpI* derivative strains of HC611 grown overnight in M9-arabinose medium were serially diluted in LB and spotted onto LB agar. The plates were incubated at 30°C and photographed after 22 h for comparison of growth phenotype. **(C)** MG1655, WJ310 (*ΔmepS ΔmepM*), WJ323 (*ΔmepS ΔmepM Δprc*), and WJ324 (*ΔmepS ΔmepM ΔnlpI*) grown overnight in M9-glucose minimal medium lacking casamino acids were diluted, spotted, incubated, and imaged in the same way as in **(B)**.

As most DD-endopeptidase genes seem to be expressed at a level comparable to that of *mepM* according to ribosome profiling data in *E. coli* (Li et al., 2014), we suspected that there could be regulatory mechanisms that inhibit these gene products from functioning in PG expansion under normal laboratory growth conditions. We reasoned that inactivation of such regulatory mechanisms might suppress the growth defect of the *mepS mepM* double mutant. To identify suppressors of the MepS^—^ MepM^—^ growth defect, a random transposon insertion library of HC611 was generated using growth medium supplemented with arabinose, and the library was selected on LB agar not supplemented with arabinose. Transposon insertions of the isolated suppressor mutants were mapped to either *prc* or *nlpI* (Figure 2A). The suppressive effect of the *prc* or *nlpI* mutation was then confirmed by constructing the *Δprc* and *ΔnlpI* derivative strains of HC611 and examining their growth phenotype (Figure 2B).

As *prc* and *nlpI* encode the proteolytic system that degrades MepS, it seemed possible that mutation of *prc* or *nlpI* might suppress the growth defect by stabilizing MepS produced by basal expression from the arabinose-inducible promoter in HC611. Thus, we also examined the effect of *prc* or *nlpI* mutation in the strain with complete deletions of *mepS* and *mepM*. As a *ΔmepS ΔmepM* double mutant strain survives in an M9-minimal medium lacking casamino acids (Singh et al., 2012; Kim et al., 2021), the *ΔmepS ΔmepM Δprc* and *ΔmepS ΔmepM ΔnlpI* triple mutant strains were generated using minimal medium. The growth defect of the *ΔmepS ΔmepM* double mutant was also suppressed by mutation of *prc* or *nlpI*, indicating that *prc* and *nlpI* are involved in the negative regulation of DD-endopeptidases other than MepS and MepM (Figure 2C).

### Suppression of the MepS^—^ MepM^—^ Growth Defect by *prc* or *nlpI* Mutation Requires MepH

**Figure 3:**
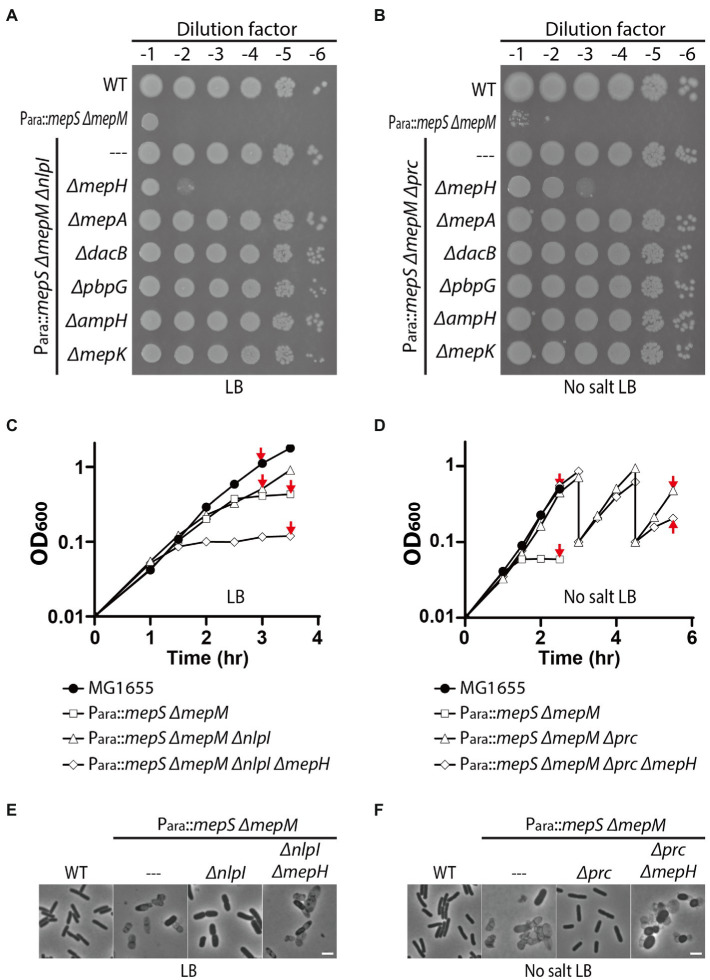
MepH is required for suppression of the *mepS mepM* mutant growth defect by inactivation of Prc or NlpI. **(A,B)** Spot dilution assay of HC611 derivative strains to identify DD-endopeptidases required for suppression of the synthetic lethality between the *mepS* and *mepM* mutations. DD-endopeptidase mutant derivatives of the *nlpI* suppressor strain (WJ76, P_ara_::*mepS*, *ΔmepM*, *ΔnlpI*) were spotted on LB agar **(A)** and the mutant derivatives of the *prc* suppressor (WJ81, P_ara_::*mepS*, *ΔmepM*, *Δprc*) were spotted on LB agar lacking NaCl **(B)**. The plates were incubated at 30°C and photographed after 22 h. **(C)** Growth curves of MG1655 (wild type), HC611 (P_ara_::*mepS*, *ΔmepM*), WJ76 (P_ara_::*mepS*, *ΔmepM*, *ΔnlpI*), WJ95 (P_ara_::*mepS*, *ΔmepM*, *ΔnlpI*, *ΔmepH*) in LB. Overnight cultures grown in M9-arabinose medium were washed and diluted in LB to an OD_600_ of 0.01. Cells were then grown with agitation at 37°C with OD600 readings taken every 30 min. **(D)** Growth curves of MG1655, HC611, WJ86 (P_ara_::*mepS*, *ΔmepM*, *Δprc*), WJ89 (P_ara_::*mepS*, *ΔmepM*, *Δprc*, *ΔmepH*) in LB lacking NaCl. The strains were grown in the same way as in (C) except that no salt LB was used instead of LB and the incubation time was prolonged to observe cell lysis. At the 3 h and 4.5 h time points, WJ86 and WJ89 cultures were diluted again to an OD_600_ of 0.1 to prevent entry into stationary phase because phenotypic difference between the cultures was more obvious when cultures were maintained in exponential phase. Shown are the representative graphs from the triplicate experiments. **(E,F)** At the indicated time points (marked with red arrows) of the growth curves presented in **(C,D)**, samples of cells were chemically fixed and imaged using phase contrast optics. Shown are the representative images from triplicate experiments. The bars equal 3 μM.

To identify DD-endopeptidase(s) whose activity is required for the survival of the *Δprc* and *ΔnlpI* suppressors of the MepS^—^ MepM^—^ growth defect, we mutated individual DD-endopeptidase genes in the *Δprc* and *ΔnlpI* derivative strains of HC611 and examined the growth phenotypes in rich medium. An obvious growth defect was observed only upon introduction of an *mepH* deletion. Inactivation of MepH caused a severe growth defect in the *ΔnlpI* suppressor on LB agar (Figure 3A). Although the *mepH* mutation did not significantly impair growth of the *Δprc* suppressor on normal LB agar, it caused a severe growth defect when LB agar lacking NaCl was used as a growth medium (Supplementary Figure 1; Figure 3B). These results indicated that MepH is the DD-endopeptidase critical for suppression of the MepS^—^ MepM^—^ growth defect by inactivation of Prc or NlpI.

We also assessed the effect of *prc*, *nlpI*, and *mepH* mutations on PG homeostasis by comparing the lysis phenotype and morphology of HC611 and its mutant derivatives. *nlpI* mutation suppressed HC611 lysis, but it did not fully restore the regular rod shape and resulted in the formation of much wider cells than wild type (Figures 3C,E). On the other hand, *prc* mutation suppressed the shape defect as well as the lysis of HC611 (Figures 3D,F). In addition, consistent with the spot dilution assay result, inactivation of MepH abrogated the suppressive effect of the *prc* and *nlpI* mutations and led to cell lysis (Figures 3E,F).

Although it was shown that *mepS*, *mepM*, and *mepH* are collectively essential for the survival of *E.coli* in minimal medium (Singh et al., 2012), our result showed that *prc* mutation suppresses this essentiality even in rich medium (Supplementary Figure 1). Thus, we further examined the *Δprc-* or *ΔnlpI-*mediated suppression of the synthetic lethality of *mepS*, *mepM*, and *mepH* mutations using various growth media (Supplementary Figure 2). In M9-minimal medium, *nlpI* mutation also suppressed the synthetic lethality. However, the *ΔnlpI* suppressor made mucoid colonies unlike the *Δprc* suppressor, indicating that *nlpI* mutation only weakly suppresses the synthetic lethality. *Prc* mutation suppressed the synthetic lethality between *mepS*, *mepM*, and *mepH* much more robustly than the *nlpI* mutation, but even the *Δprc* suppressor was not able to survive if LB agar lacking NaCl was used as the growth medium. Thus, these results indicated that *mepS*, *mepM*, and *mepH* are indeed the DD-endopeptidases that play a major role in PG assembly in *E.coli*, although it is unclear how *prc* mutation suppresses the synthetic lethality much more strongly than *nlpI* mutation.

### Overexpression of *prc* or *nlpI* Phenocopies *mepH* Deletion in the MepS^—^ MepM^—^ Strain

**Figure 4:**
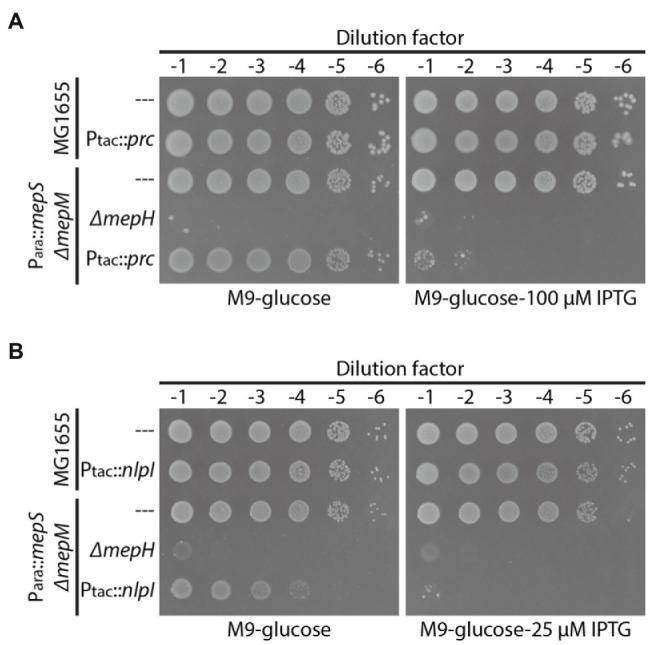
Overexpression of *prc* or *nlpI* causes synthetic lethality with *mepS* and *mepM* mutations in minimal medium. **(A)** MG1655(attHKpWJ90, P_tac_::empty), MG1655 (attHKpWJ186, P_tac_::*prc*), HC611[P_ara_::*mepS*, *ΔmepM*](attHKpWJ90), WJ283(P_ara_::*mepS*, *ΔmepM*, *ΔmepH*;attHKpWJ90), HC611(attHKpWJ186) strains grown overnight in M9-arabinose medium were diluted in M9-glucose lacking casamino acids (CAA) and spotted on M9-glucose (no CAA) agar lacking or containing 100 μM IPTG for induction of *prc* expression. The plates were photographed after incubation for 40 h at 37°C. **(B)** MG1655(attHKpWJ90), MG1655 (attHKpWJ72, P_tac_::*nlpI*), HC611(attHKpWJ90), WJ283(attHKpWJ90), HC611(attHKpWJ72) strains were grown, diluted, and spotted in the same way as in **(A)** except that 25 μM IPTG was used for induction of *nlpI* expression. The plates were photographed after incubation for 40 h at 30°C.

Requirement of MepH for *Δprc-* or *ΔnlpI*-mediated suppression of the MepS^—^ MepM^—^ growth defect suggested that MepH might also be negatively controlled by Prc and NlpI. Thus, we assessed the effects of *nlpI* or *prc* overexpression on the survival of the MepS^—^ MepM^—^ strain in minimal medium. If MepH is under the proteolytic control by Prc and NlpI, overexpression of *prc* or *nlpI* might accelerate MepH degradation and cause synthetic lethality with the *mepS* and *mepM* mutations in minimal medium. Consistent with this idea, overexpression of *prc* or *nlpI* in HC611 caused a severe growth defect on M9 glucose agar similar to that caused by *mepH* mutation (Figures 4A,B). Importantly, overexpression of *prc* or *nlpI* did not cause a similar growth defect in the wild-type strain, indicating that the growth defect arises due to synthetic lethality with inactivation of MepS and MepM rather than a general toxicity of *prc* or *nlpI* overexpression.

### MepH Level Significantly Increases in the ***mepS mepM*** Mutant

**Figure 5:**
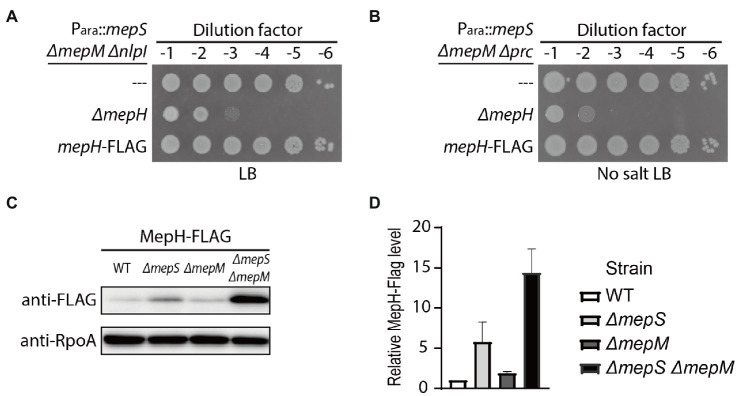
MepH levels increase in the *ΔmepS ΔmepM* strain. **(A,B)** Spot dilution assay to assess the functionality of MepH-FLAG. **(A)** The growth phenotype of WJ126 (P_ara_::*mepS ΔmepM ΔnlpI mepH*-FLAG) was compared with that of WJ76 (P_ara_::*mepS ΔmepM ΔnlpI*) and WJ95 (P_ara_::*mepS ΔmepM ΔnlpI ΔmepH*) by incubation on LB agar for 22 h at 30°C. **(B)** The growth phenotype of WJ125 (P_ara_::*mepS ΔmepM Δprc mepH*-FLAG) was compared with that of WJ81 (P_ara_::*mepS ΔmepM Δprc*) and WJ89 (P_ara_::*mepS ΔmepM Δprc ΔmepH*) by incubation on LB agar lacking salt for 22 h at 30°C. **(C)** MepH-FLAG levels in the *ΔmepS* and *ΔmepM* mutants. Strains with MepH tagged with 3X FLAG at the native chromosomal locus, WJ203, WJ373 (*ΔmepS*), WJ374 (*ΔmepM*), and WJ309 (*ΔmepS ΔmepM*), were grown overnight in M9 glucose medium lacking casamino acids. The cultures were washed and diluted in LB to an OD_600_ of 0.05. Cells were then grown with agitation at 30°C and harvested around an OD_600_ of 0.5. Equivalent amounts of whole-cell lysates of each strain were used for immunoblotting with anti-FLAG and anti-RpoA antibodies. Shown are the representative images from triplicate experiments. **(D)** The band intensities of the Western blot in **(C)** were quantified using ImageJ software and the relative MepH-Flag levels are shown after normalization with RpoA signals. Error bars represent the standard deviation from triplicate measurements.

To examine the effects of various DD-endopeptidase-related mutations on cellular MepH levels, we fused a 3X FLAG tag to the C-terminus of MepH at the native chromosomal locus. The resulting MepH-FLAG appeared functional, since strains with the *mepH*-FLAG fusion showed growth phenotypes similar to strains with untagged *mepH* (Figures 5A,B). As the immunoblot signal for MepH-FLAG was relatively weak, we looked for a strain where the MepH-FLAG signal could be readily detected. We suspected that MepH levels might increase in the *mepS mepM* strain because MepH activity is essential for the survival of the *mepS mepM* strain. Indeed, MepH-FLAG levels slightly increased in the *mepS* mutant and increased more than 10-fold in the *ΔmepS ΔmepM* strain compared with the MepH level in the wild-type strain, suggesting that the low DD-endopeptidase activity somehow induces *mepH* expression or delays MepH degradation in the *ΔmepS ΔmepM* strain (Figures 5C,D).

### MepH Is Under the Proteolytic Control of Prc and NlpI

**Figure 6:**
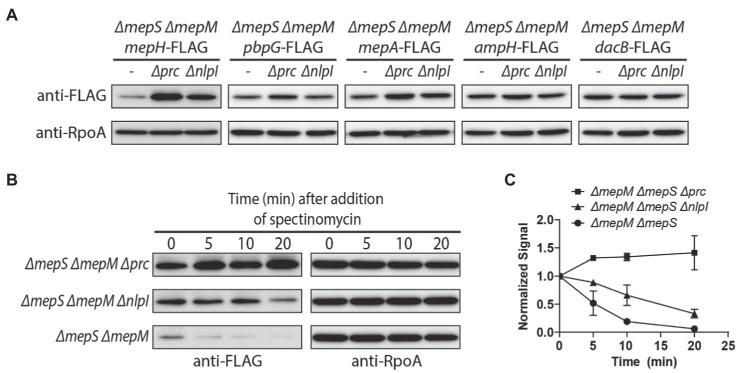
Prc and NlpI are involved in the negative regulation of MepH levels. **(A)** Comparison of DD-endopeptidase levels in the *ΔmepS ΔmepM*, *ΔmepS ΔmepM Δprc, and ΔmepS ΔmepM ΔnlpI* strains. The *ΔmepS ΔmepM* strains with DD-endopeptidase tagged with 3X FLAG and their *Δprc* and *ΔnlpI* derivatives were grown overnight in M9 glucose lacking casamino acids. The cells were washed and diluted in LB to an OD600 of 0.05. When the cultures reached an OD_600_ of 0.5, cells were harvested by centrifugation, resuspended in Laemmli buffer, and used for immunoblotting. **(B,C)** *In vivo* degradation assay of MepH-Flag. WJ309 (*ΔmepS ΔmepM mepH-FLAG*), WJ199 (*ΔmepS ΔmepM Δprc mepH-FLAG*), and WJ200 (*ΔmepS ΔmepM ΔnlpI mepH-FLAG*) were grown to an OD_600_ of 0.5 in LB, as described in **(A)**. Spectinomycin was added to each culture to a final concentration of 500 μg/ml to block protein synthesis and aliquots were collected at the indicated time points for immunoblotting. The anti-FLAG signal of each sample was normalized to the anti-RpoA signal. Each experiment was performed in triplicates and the representative images are shown. **(C)** The normalized signal at the time of spectinomycin addition was set as 1 and the change in signal intensity at each time point was plotted for each strain. Error bars represent the standard deviation from triplicate measurements.

As MepH levels are higher in the *ΔmepS ΔmepM* strain, and it is also relevant to assess the effects of *prc* and *nlpI* mutations in the strain where we can observe suppression of the growth defect, we compared MepH levels in the *ΔmepS ΔmepM* strain background (Figure 6A). The *prc* and *nlpI* mutations caused a significant increase in MepH levels. The MepH-FLAG signal increased more than 5-fold by the *prc* mutation and about 3.5-fold by the *nlpI* mutation, indicating that MepH is negatively controlled by the Prc/NlpI proteolytic system. We also compared the levels of other DD-endopeptidases between the *ΔmepS ΔmepM* strain and its *Δprc* and *ΔnlpI* derivatives by fusing the FLAG tag to the C-terminus of each gene at their native chromosomal loci (Figure 6A). The levels of PBP7 (*pbpG*), MepA, and AmpH also increased slightly in the *Δprc* and *ΔnlpI* derivatives, but the increase was less than 2-fold, indicating that Prc and NlpI are not significantly involved in the negative regulation of these DD-endopeptidases.

Next, we monitored MepH levels in the *ΔmepS ΔmepM* strain and its *prc* and *nlpI* mutant derivatives after inhibition of protein synthesis to test if Prc and NlpI are involved in MepH degradation (Figures 6B,C). While MepH levels rapidly decreased in the *ΔmepS ΔmepM* strain, it did not noticeably decrease in its *Δprc* derivative strain over the same time interval. MepH levels decreased slightly in the *ΔnlpI* derivative strain, but the rate of decrease was lower than the parental strain. These results suggested that the higher MepH levels in the *prc* and *nlpI* mutants are due to reduced degradation rather than increased production, indicating that Prc and NlpI are indeed involved in the proteolytic control of MepH. The slow decrease of MepH levels in the *nlpI* mutant suggested that MepH can be degraded by Prc in an NlpI-independent way.

**Figure 7:**
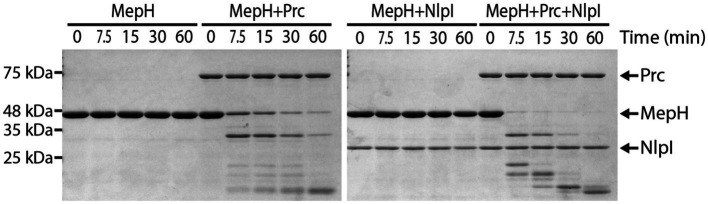
Prc degrades MepH and this degradation is enhanced by NlpI. Time course experiment for MepH degradation in the presence or absence of Prc and NlpI. Four different reactions that contained the indicated proteins were prepared in PBS buffer and incubated at 37°C. Note that His6-SUMO-tagged MepH (H-SUMO-MepH) was used instead of MepH because untagged MepH was not soluble. H-SUMO-MepH is labeled as MepH in this figure for convenience. The concentrations of each protein added to the reactions were 5 μM (MepH), 1 μM (Prc), and 2 μM (NlpI). Aliquots were taken from each reaction at the indicated time points, mixed with Laemmli sample buffer, and boiled. Samples were separated on 12% SDS-PAGE gels and the gels were stained with Coomassie brilliant blue. Positions of molecular weight markers are indicated on the left and positions of the proteins used in the assay are indicated with arrows on the right.

To determine if MepH is a direct substrate of the Prc/NlpI proteolytic system, we purified each protein and tested if MepH is degraded in the presence of Prc and NlpI (Figure 7). We used His6-SUMO-tagged MepH instead of MepH because untagged MepH was not soluble in our experimental conditions. His6-SUMO-tagged MepH will be referred to as MepH for convenience. MepH was stable when incubated alone or with NlpI, but was degraded rapidly in the presence of Prc. The degradation was further accelerated when NlpI was also added to the reaction. Interestingly, the degradation of MepH by Prc was initially fast even without NlpI, but slowed down when MepH concentration was reduced in the reaction lacking NlpI. On the other hand, MepH was rapidly degraded to an undetectable level in the reaction that contained both Prc and NlpI, suggesting NlpI is indeed involved in MepH degradation, especially when MepH concentration is low.

## Discussion

Cell wall expansion in Gram-negative bacteria requires DD-endopeptidase activity that cleaves the PG network for insertion of newly synthesized PG material, and this activity needs to be tightly regulated to prevent self-degradation of the cell wall. The potential lethality of uncontrolled DD-endopeptidase activity is exemplified by the fact that the predatory bacterium *Bdellovibrio bacteriovorus* secretes DD-endopeptidases to degrade the PG of its prey Gram-negative bacteria (Lerner et al., 2012). However, it is still not clearly understood how Gram-negative bacteria regulate their DD-endopeptidase activity to achieve PG expansion and remodeling without harming their cell wall. The best-studied regulatory mechanism of DD-endopeptidases is the proteolytic control of these enzymes by periplasmic protease-adaptor pairs (Singh et al., 2015; Srivastava et al., 2018). In *E.coli*, MepS was shown to be degraded by the protease Prc and the lipoprotein adaptor NlpI (Singh et al., 2015; Su et al., 2017). Recently, MepM was also shown to be degraded by Prc, but in an NlpI-independent manner (Kim et al., 2021). In this study, we discovered that MepH is also negatively controlled by Prc and NlpI, showing that the DD-endopeptidases playing a major role in cell wall expansion under normal physiological conditions are controlled by a common protease.

During the characterization of *prc* and *nlpI* mutations that suppress the MepS^—^ MepM^—^ growth defect, we discovered that MepH is required for this suppression and MepH levels increased in the *prc* or *nlpI* mutant. MepH accumulated to a higher level in the *prc* mutant than in the *nlpI* mutant. Consistent with the higher level of MepH in the *prc* mutant than the *nlpI* mutant, inactivation of Prc suppressed the MepS^—^ MepM^—^ growth defect much more strongly than the *nlpI* mutation. Tracking MepH levels after inhibition of protein synthesis revealed that MepH degradation is delayed in both the *prc* and *nlpI* mutants, but the degradation is much slower in the *prc* mutant than in the *nlpI* mutant. This result suggested that MepH can be degraded by Prc in an NlpI-independent manner although the degradation is accelerated in the presence of NlpI. An *in vitro* MepH degradation assay indicated that the NlpI-independent degradation of MepH by Prc can occur without an adaptor, rather than requiring an alternative adaptor between MepH and Prc.

Although the higher MepH level in the *Δprc* strain is consistent with the stronger suppressive effect of the *prc* mutation, MepH levels could not completely explain the difference in the suppressive effect between the *prc* and *nlpI* mutations. The *mepS mepM prc* triple mutant could still survive when *mepH* is deleted, while survival of the *mepS mepM nlpI* strain was completely dependent on MepH in LB (Supplementary Figure 2). This phenotypic difference can be explained if the level or activity of other DD-endopeptidases is higher in the *Δprc* strain than in the *ΔnlpI* strain. However, the level of other DD-endopeptidases, PBP7, MepA, and AmpH, was not much higher in the *prc* mutant than in the *nlpI* mutant, failing to provide support for this idea (Figure 6A). Alternatively, the phenotypic difference could occur if NlpI has a Prc-independent role in supporting PG assembly in addition to its function as an adaptor between Prc and DD-endopeptidases. Then, *nlpI* mutation would exert undermining as well as enhancing effects on PG assembly and thus would not be able to efficiently suppress the growth defect caused by DD-endopeptidase mutations. Although most of the known functions of NlpI are related to the proteolytic activity of Prc (Singh et al., 2015; Yakhnina and Bernhardt, 2020), NlpI was recently proposed to affect the activity of other types of PG remodeling enzymes (Banzhaf et al., 2020). Some of these interactions might affect PG assembly in an Prc-independent manner.

In this study, we observed that MepH level increases significantly by inactivation of major DD-endopeptidases MepS and MepM. This result indicates that there are mechanisms that sense the deficiency of DD-endopeptidase activity and upregulate MepH level to maintain cell wall homeostasis. Upregulation of PG endopeptidase expression upon inactivation of PG endopeptidases was also recently reported in *B. subtilis*, where the WalK/WalR two component system regulates transcription of DL-endopeptidases by sensing the cleavage products generated by these enzymes (Dobihal et al., 2019). It remains to be determined whether elevation of MepH level in the DD-endopeptidase mutant is mediated by similar transcriptional control or other types of regulatory mechanism, such as modulating MepH proteolysis by Prc.

It is important to understand how the activities of PG synthetic and hydrolytic enzymes are coordinated to achieve PG assembly without causing lethal breaches in the cell wall. Although much remains to be understood regarding the mechanisms that regulate DD-endopeptidase activity essential for PG expansion, this work shows that a proteolytic system consisting of Prc and NlpI negatively controls MepH.

## Data Availability Statement

The datasets presented in this study can be found in online repositories. The names of the repository/repositories and accession number(s) can be found in the article/Supplementary Material.

## Author Contributions

HC and W-JJ designed the research and analyzed the data. W-JJ performed the research. HC wrote the paper. All authors contributed to the article and approved the submitted version.

## Funding

This study was supported by the National Research Foundation of KOREA (NRF-2019R1A2C1002648).

## Conflict of Interest

The authors declare that the research was conducted in the absence of any commercial or financial relationships that could be construed as a potential conflict of interest.

## Publisher’s Note

All claims expressed in this article are solely those of the authors and do not necessarily represent those of their affiliated organizations, or those of the publisher, the editors and the reviewers. Any product that may be evaluated in this article, or claim that may be made by its manufacturer, is not guaranteed or endorsed by the publisher.

## Supplementary Material

The Supplementary Material for this article can be found online at: https://www.frontiersin.org/articles/10.3389/fmicb.2022.878049/full#supplementary-material

## References

[ref1] Baba T. Ara T. Hasegawa M. Takai Y. Okumura Y. Baba M.. (2006). Construction of Escherichia coli K-12 in-frame, single-gene knockout mutants: the Keio collection. Mol. Syst. Biol. 2:20060008. doi: 10.1038/msb4100050, PMID: 16738554 PMC1681482

[ref2] Banzhaf M. Yau H. C. Verheul J. Lodge A. Kritikos G. Mateus A.. (2020). Outer membrane lipoprotein NlpI scaffolds peptidoglycan hydrolases within multi-enzyme complexes in Escherichia coli. EMBO J. 39:e102246. doi: 10.15252/embj.2019102246, PMID: 32009249 PMC7049810

[ref3] Bernhardt T. G. Boer P. A. J. D. (2003). The Escherichia coli amidase AmiC is a periplasmic septal ring component exported via the twin-arginine transport pathway. Mol. Microbiol. 48, 1171–1182. doi: 10.1046/j.1365-2958.2003.03511.x, PMID: 12787347 PMC4428285

[ref4] Chodisetti P. K. Reddy M. (2019). Peptidoglycan hydrolase of an unusual cross-link cleavage specificity contributes to bacterial cell wall synthesis. Proc. National Acad. Sci. 116, 7825–7830. doi: 10.1073/pnas.1816893116, PMID: 30940749 PMC6475434

[ref5] Datsenko K. A. Wanner B. L. (2000). One-step inactivation of chromosomal genes in Escherichia coli K-12 using PCR products. Proc. National Acad. Sci. 97, 6640–6645. doi: 10.1073/pnas.120163297, PMID: 10829079 PMC18686

[ref6] Dobihal G. S. Brunet Y. R. Flores-Kim J. Rudner D. Z. (2019). Homeostatic control of cell wall hydrolysis by the WalRK two-component signaling pathway in Bacillus subtilis. elife 8:e52088. doi: 10.7554/elife.52088, PMID: 31808740 PMC7299342

[ref7] Dörr T. Cava F. Lam H. Davis B. M. Waldor M. K. (2013). Substrate specificity of an elongation-specific peptidoglycan endopeptidase and its implications for cell wall architecture and growth of vibrio cholerae. Mol. Microbiol. 89, 949–962. doi: 10.1111/mmi.12323, PMID: 23834664 PMC3769093

[ref8] Ferrières L. Hémery G. Nham T. Guérout A.-M. Mazel D. Beloin C.. (2010). Silent mischief: bacteriophage mu insertions contaminate products of *Escherichia coli* random mutagenesis performed using suicidal transposon delivery plasmids mobilized by broad-host-range RP4 conjugative machinery. J. Bacteriol. 192, 6418–6427. doi: 10.1128/jb.00621-10, PMID: 20935093 PMC3008518

[ref9] Guyer M. S. Reed R. R. Steitz J. A. Low K. B. (1981). Identification of a sex-factor-affinity site in *E. coli* as. Cold Spring Harb. Sym. 45, 135–140. doi: 10.1101/sqb.1981.045.01.022 6271456

[ref10] Hashimoto M. Ooiwa S. Sekiguchi J. (2012). Synthetic lethality of the lytE cwlO genotype in Bacillus subtilis is caused by lack of d, l-Endopeptidase activity at the lateral Cell Wall. J. Bacteriol. 194, 796–803. doi: 10.1128/jb.05569-11, PMID: 22139507 PMC3272963

[ref11] Johnson J. E. Lackner L. L. Hale C. A. Boer P. A. J. (2004). ZipA is required for targeting of D MinC/DicB, but not D MinC/MinD, complexes to Septal ring assemblies in *Escherichia coli*. J. Bacteriol. 186, 2418–2429. doi: 10.1128/jb.186.8.2418-2429.2004, PMID: 15060045 PMC412171

[ref12] Kim Y. J. Choi B. J. Park S. H. Lee H. B. Son J. E. Choi U.. (2021). Distinct amino acid availability-dependent regulatory mechanisms of MepS and MepM levels in Escherichia coli. Front. Microbiol. 12:677739. doi: 10.3389/fmicb.2021.677739, PMID: 34276609 PMC8278236

[ref13] Lerner T. R. Lovering A. L. Bui N. K. Uchida K. Aizawa S.-I. Vollmer W.. (2012). Specialized peptidoglycan hydrolases sculpt the intra-bacterial niche of predatory Bdellovibrio and increase population fitness. PLoS Pathog. 8:e1002524. doi: 10.1371/journal.ppat.1002524, PMID: 22346754 PMC3276566

[ref14] Li G.-W. Burkhardt D. Gross C. Weissman J. S. (2014). Quantifying absolute protein synthesis rates reveals principles underlying allocation of cellular resources. Cell 157, 624–635. doi: 10.1016/j.cell.2014.02.033, PMID: 24766808 PMC4006352

[ref15] Marblestone J. G. Edavettal S. C. Lim Y. Lim P. Zuo X. Butt T. R. (2006). Comparison of SUMO fusion technology with traditional gene fusion systems: enhanced expression and solubility with SUMO. Protein Sci. Publ. Protein Soc. 15, 182–189. doi: 10.1110/ps.051812706, PMID: 16322573 PMC2242369

[ref16] Mossessova E. Lima C. D. (2000). Ulp1-SUMO crystal structure and genetic analysis reveal conserved interactions and a regulatory element essential for cell growth in yeast. Mol. Cell 5, 865–876. doi: 10.1016/s1097-2765(00)80326-3, PMID: 10882122

[ref17] Murphy S. G. Alvarez L. Adams M. C. Liu S. Chappie J. S. Cava F.. (2019). Endopeptidase regulation as a novel function of the Zur-dependent zinc starvation response. MBio 10:e02620-18. doi: 10.1128/mbio.02620-18, PMID: 30782657 PMC6381278

[ref18] Murphy S. G. Murtha A. N. Zhao Z. Alvarez L. Diebold P. Shin J.-H.. (2021). Class A penicillin-binding protein-mediated Cell Wall synthesis promotes structural integrity during peptidoglycan Endopeptidase insufficiency in vibrio cholerae. MBio 12:e03596-20. doi: 10.1128/mbio.03596-20, PMID: 33824203 PMC8092314

[ref19] Singh S. K. Parveen S. SaiSree L. Reddy M. (2015). Regulated proteolysis of a cross-link–specific peptidoglycan hydrolase contributes to bacterial morphogenesis. Proc. National Acad. Sci. 112, 10956–10961. doi: 10.1073/pnas.1507760112, PMID: 26283368 PMC4568209

[ref20] Singh S. K. SaiSree L. Amrutha R. N. Reddy M. (2012). Three redundant murein endopeptidases catalyse an essential cleavage step in peptidoglycan synthesis of Escherichia coli K12. Mol. Microbiol. 86, 1036–1051. doi: 10.1111/mmi.12058, PMID: 23062283

[ref21] Srivastava D. Seo J. Rimal B. Kim S. J. Zhen S. Darwin A. J. (2018). A Proteolytic complex targets multiple Cell Wall hydrolases in *Pseudomonas aeruginosa*. MBio 9:e00972-18. doi: 10.1128/mbio.00972-18, PMID: 30018106 PMC6050968

[ref22] Su M.-Y. Som N. Wu C.-Y. Su S.-C. Kuo Y.-T. Ke L.-C.. (2017). Structural basis of adaptor-mediated protein degradation by the tail-specific PDZ-protease Prc. Nat. Commun. 8:1516. doi: 10.1038/s41467-017-01697-9, PMID: 29138488 PMC5686067

[ref23] Vollmer W. (2012). Bacterial growth does require peptidoglycan hydrolases. Mol. Microbiol. 86, 1031–1035. doi: 10.1111/mmi.12059 23066944

[ref24] Yakhnina A. A. Bernhardt T. G. (2020). The Tol-pal system is required for peptidoglycan-cleaving enzymes to complete bacterial cell division. Proc National Acad Sci 117, 6777–6783. doi: 10.1073/pnas.1919267117, PMID: 32152098 PMC7104345

