# Supplementary material for: A Cell Wall Hydrolase MepH Is Negatively Regulated by Proteolysis Involving Prc and NlpI in Escherichia coli
Source: Front Microbiol. 2022 Mar 28;13:878049. doi: 10.3389/fmicb.2022.878049 (PMC8996183; doi:10.3389/fmicb.2022.878049)
Supplement: Supplementary file 1 [file Data_Sheet_1.docx]

**SUPPLEMENTARY MATERIAL FOR:**

**A cell wall hydrolase MepH is negatively regulated by proteolysis involving Prc and NlpI in *Escherichia coli*.**

Wook-Jong Jeon^1^ and Hongbaek Cho^1^*

^1^Department of Biological Sciences, College of Natural Sciences, Sungkyunkwan University,

*To whom correspondence should be addressed.

Hongbaek Cho, Ph.D.

Sungkyunkwan University

Department of Biological Sciences

Suwon 16419, Republic of Korea

e-mail: [hongbaek@skku.edu](mailto:hongbaek@skku.edu)


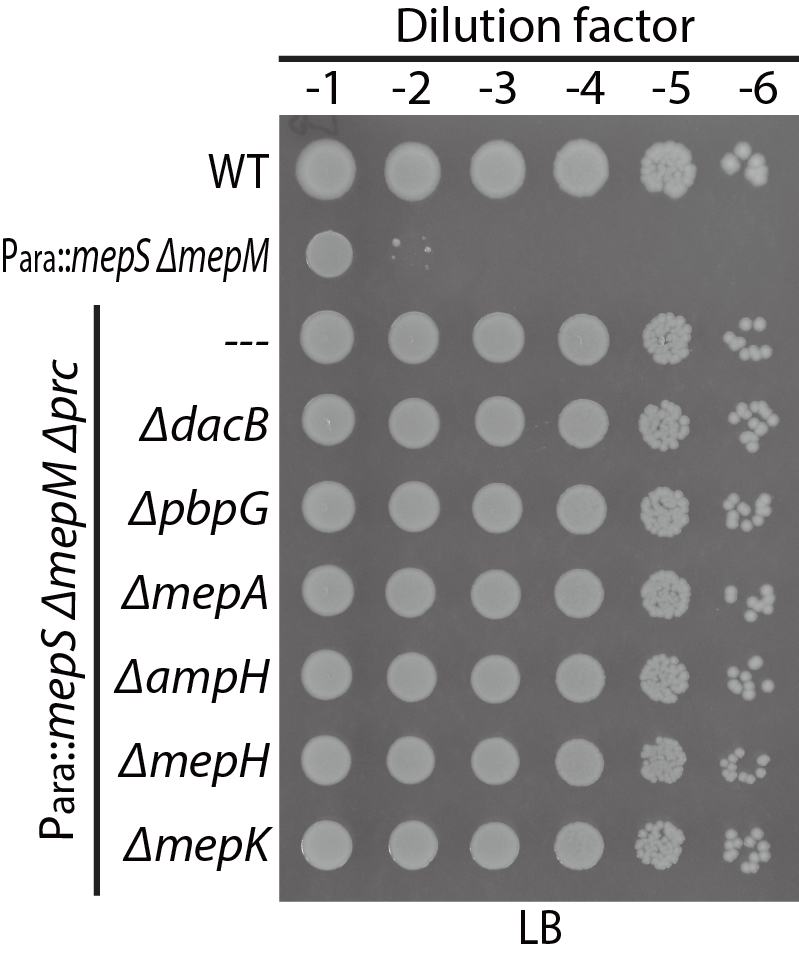


**Supplementary Figure 1. Spot dilution assay of DD-endopeptidase mutant derivative strains of HC611 on LB agar.** MG1655, HC611 (P_ara_::*mepS*, *ΔmepM*), WJ81 (P_ara_::*mepS*, *ΔmepM*, *Δprc*), and the DD-endopeptidase mutant derivatives of WJ81 were grown overnight in M9-arabinose medium, serially diluted in LB, and the dilutions spotted on LB-agar as in Fig. 3. The plates were incubated at 30 °C and photographed after 22 hours.


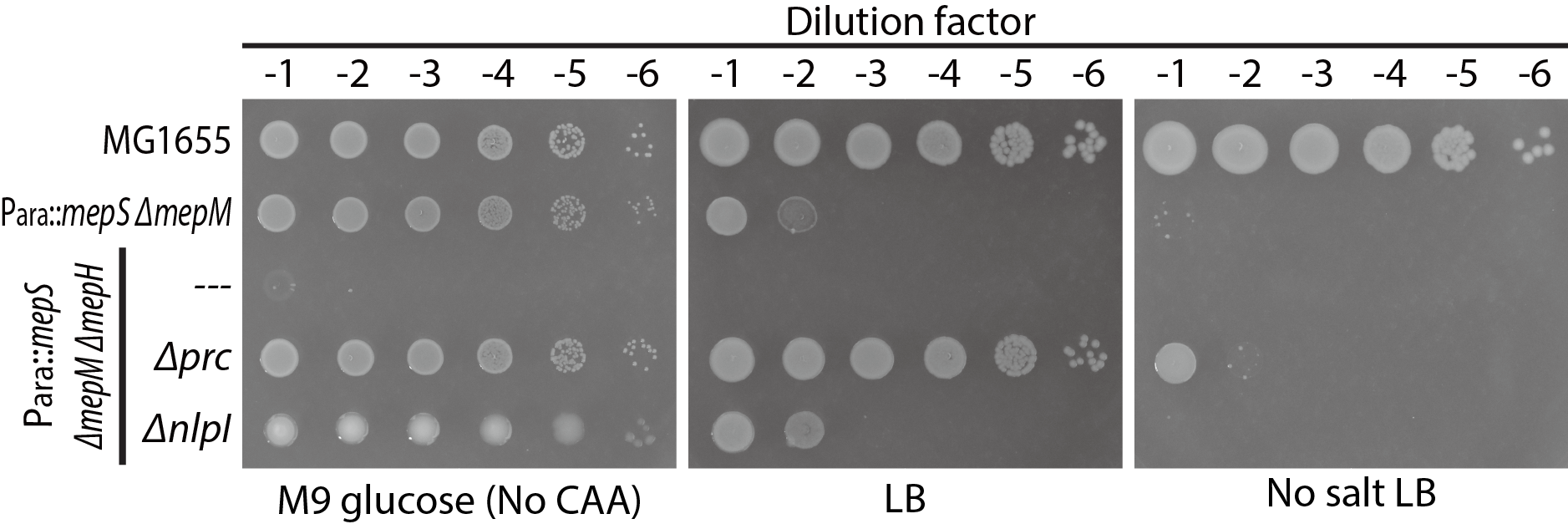


**Supplementary Figure 2. Inactivation of Prc or NlpI suppresses the collective essentiality of *mepS*, *mepM*, and *mepH*.** To assess suppression of the synthetic lethality between *mepS*, *mepM*, and *mepH* by inactivation of Prc or NlpI, MG1655, HC611 (P_ara_::*mepS* *ΔmepM*), WJ283 (P_ara_::*mepS* *ΔmepM ΔmepH*), WJ89 (P_ara_::*mepS* *ΔmepM ΔmepH Δprc*), and WJ95 (P_ara_::*mepS* *ΔmepM ΔmepH ΔnlpI*) strains were grown overnight in M9-arabinose medium. The overnight cultures were 10-fold serially diluted in M9-glucose medium lacking casamino acids, LB medium, or LB medium lacking NaCl, and spotted on the corresponding solid agar media. Then, the agar plates were incubated at 30 °C. The LB and no salt LB agar plates were photographed after incubation for 22 hours and the M9-glucose agar plate after incubation for 40 hours.

**Supplementary Table 1. Strains used in this study**

| Strain | Genotype | Source/  Reference |
| --- | --- | --- |
| MG1655 | *rph ilvG rfb-50* | (Guyer et al., 1981) |
| TB10 | *rph1 ilvG rfb-50 λ*Δ*cro-bio nad*::*Tn10* | (Johnson et al., 2004) |
| TB28 | MG1655 Δ*lacIZYA*<>*frt* | (Bernhardt and Boer, 2004) |
| DH5α(*λpir*) | F- *endA1 hsdR17* (*r^-^m^+^*) *supE44 thi-1 recA1 gyrA relA1* Δ(*lacZYA-argF*)*_u189_ Φ80lacZ*Δ*M15 λpir* | (Pal et al., 2005) |
| MFD*pir* | *RP4-2-Tc::[ΔMu1::aac(3)IV-ΔaphA-Δnic35-ΔMu2::zeo] ΔdapA::(erm-pir) ΔrecA* | (Ferrières et al., 2010) |
| BL21(DE3)/pLysS | *ompT* rB− mB− (P_lac_*UV5*::T7*gene*1)  pLysS[*T7p20 ori*_p15A_](Cm^R^) | Lab collection |
| HC611 | *rph1 ilvG rfb-50* Δ*lacIZYA<>frt* Δ*mepM<>frt* P_ara_::*mepS* | This study |
| WJ76 | *rph1 ilvG rfb-50* Δ*lacIZYA<>frt* Δ*mepM<>frt* P_ara_::*mepS* Δ*nlpI<>frt* | This study |
| WJ81 | *rph1 ilvG rfb-50* Δ*lacIZYA<>frt* Δ*mepM<>frt* P_ara_::*mepS* Δ*prc*<>*frt* | This study |
| WJ85 | *rph1 ilvG rfb-50* Δ*lacIZYA<>frt* Δ*mepM<>frt* P_ara_::*mepS* Δ*prc*<>*frt* Δ*dacB*::*aph* | This study |
| WJ86 | *rph1 ilvG rfb-50* Δ*lacIZYA<>frt* Δ*mepM<>frt* P_ara_::*mepS* Δ*prc*<>*frt* Δ*pbpG*::*aph* | This study |
| WJ87 | *rph1 ilvG rfb-50* Δ*lacIZYA<>frt* Δ*mepM<>frt* P_ara_::*mepS* Δ*prc*<>*frt* Δ*mepA*::*aph* | This study |
| WJ88 | *rph1 ilvG rfb-50* Δ*lacIZYA<>frt* Δ*mepM<>frt* P_ara_::*mepS* Δ*prc*<>*frt* Δ*ampH*::*aph* | This study |
| WJ89 | *rph1 ilvG rfb-50* Δ*lacIZYA<>frt* Δ*mepM<>frt* P_ara_::*mepS* Δ*prc*<>*frt* Δ*mepH*::*aph* | This study |
| WJ91 | *rph1 ilvG rfb-50* Δ*lacIZYA<>frt* Δ*mepM<>frt* P_ara_::*mepS* Δ*nlpI<>frt* Δ*dacB*::*aph* | This study |
| WJ92 | *rph1 ilvG rfb-50* Δ*lacIZYA<>frt* Δ*mepM<>frt* P_ara_::*mepS* Δ*nlpI<>frt* Δ*pbpG*::*aph* | This study |
| WJ93 | *rph1 ilvG rfb-50* Δ*lacIZYA<>frt* Δ*mepM<>frt* P_ara_::*mepS* Δ*nlpI<>frt* Δ*mepA*::*aph* | This study |
| WJ94 | *rph1 ilvG rfb-50* Δ*lacIZYA<>frt* Δ*mepM<>frt* P_ara_::*mepS* Δ*nlpI<>frt* Δ*ampH*::*aph* | This study |
| WJ95 | *rph1 ilvG rfb-50* Δ*lacIZYA<>frt* Δ*mepM<>frt* P_ara_::*mepS* Δ*nlpI<>frt* Δ*mepH*::*aph* | This study |
| WJ124 | *rph1 ilvG rfb-50* Δ*lacIZYA<>frt* Δ*mepM<>frt* P_ara_::*mepS mepH-FLAG*::*aph* | This study |
| WJ125 | rph1 ilvG rfb-50 ΔlacIZYA<>frt ΔmepM<>frt P_ara_::*mepS* Δ*prc*<>frt *mepH-FLAG*::*aph* | This study |
| WJ126 | *rph1 ilvG rfb-50* Δ*lacIZYA<>frt* Δ*mepM<>frt* P_ara_::*mepS* Δ*nlpI<>frt mepH-FLAG*::*aph* | This study |
| WJ199 | *rph1 ilvG rfb-50* Δ*mepS<>frt* Δ*prc*<>*frt mepH-FLAG*<>*frt* Δ*mepM*::*aph* | This study |
| WJ200 | *rph1 ilvG rfb-50* Δ*mepS<>frt* Δ*nlpI*<>*frt mepH-FLAG*<>*frt* Δ*mepM*::*aph* | This study |
| WJ203 | *rph1 ilvG rfb-50 mepH-FLAG*<>*frt* | This study |
| WJ283 | *rph1 ilvG rfb-50* Δ*lacIZYA<>frt* Δ*mepM<>frt* P_ara_::*mepS* Δ*mepH*::*aph* | This study |
| WJ309 | *rph1 ilvG rfb-50 mepH-FLAG<>frt* Δ*mepS<>frt* Δ*mepM*::*aph* | This study |
| WJ310 | *rph1 ilvG rfb-50* Δ*mepS<>frt* Δ*mepM<>frt* | This study |
| WJ321 | *rph1 ilvG rfb-50* Δ*lacIZYA<>frt* Δ*mepM<>frt* P_ara_::*mepS* Δ*prc*<>*frt* Δ*mepK*::*aph* | This study |
| WJ322 | *rph1 ilvG rfb-50* Δ*lacIZYA<>frt* Δ*mepM<>frt* P_ara_::*mepS* Δ*nlpI<>frt* Δ*mepK*::*aph* | This study |
| WJ323 | *rph1 ilvG rfb-50* Δ*mepS<>frt* Δ*mepM<>frt* Δ*prc*::*aph* | This study |
| WJ324 | *rph1 ilvG rfb-50* Δ*mepS<>frt* Δ*mepM<>frt* Δ*nlpI*::*aph* | This study |
| WJ344 | *rph1 ilvG rfb-50* Δ*mepS<>frt* Δ*mepM<>frt mepK-FLAG*::*aph* | This study |
| WJ345 | *rph1 ilvG rfb-50* Δ*mepS<>frt* Δ*mepM<>frt dacB-FLAG*::*aph* | This study |
| WJ346 | *rph1 ilvG rfb-50* Δ*mepS<>frt* Δ*mepM<>frt pbpG-FLAG*::*aph* | This study |
| WJ347 | *rph1 ilvG rfb-50* Δ*mepS<>frt* Δ*mepM<>frt mepA-FLAG*::*aph* | This study |
| WJ348 | *rph1 ilvG rfb-50* Δ*mepS<>frt* Δ*mepM<>frt ampH-FLAG*::*aph* | This study |
| WJ350 | *rph1 ilvG rfb-50* Δ*mepS<>frt* Δ*mepM<>frt* Δ*prc*::*cat mepK-FLAG*::*aph* | This study |
| WJ351 | *rph1 ilvG rfb-50* Δ*mepS<>frt* Δ*mepM<>frt* Δ*prc*::*cat dacB-FLAG*::*aph* | This study |
| WJ352 | *rph1 ilvG rfb-50* Δ*mepS<>frt* Δ*mepM<>frt* Δ*prc*::*cat pbpG-FLAG*::*aph* | This study |
| WJ353 | *rph1 ilvG rfb-50* Δ*mepS<>frt* Δ*mepM<>frt* Δ*prc*::*cat mepA-FLAG*::*aph* | This study |
| WJ354 | *rph1 ilvG rfb-50* Δ*mepS<>frt* Δ*mepM<>frt* Δ*prc*::*cat ampH-FLAG*::*aph* | This study |
| WJ356 | *rph1 ilvG rfb-50* Δ*mepS<>frt* Δ*mepM<>frt* Δ*nlpI::cat mepK-FLAG*::*aph* | This study |
| WJ357 | *rph1 ilvG rfb-50* Δ*mepS<>frt* Δ*mepM<>frt* Δ*nlpI::cat dacB-FLAG*::*aph* | This study |
| WJ358 | *rph1 ilvG rfb-50* Δ*mepS<>frt* Δ*mepM<>frt* Δ*nlpI::cat pbpG-FLAG*::*aph* | This study |
| WJ359 | *rph1 ilvG rfb-50* Δ*mepS<>frt* Δ*mepM<>frt* Δ*nlpI::cat mepA-FLAG*::*aph* | This study |
| WJ360 | *rph1 ilvG rfb-50* Δ*mepS<>frt* Δ*mepM<>frt* Δ*nlpI::cat ampH-FLAG*::*aph* | This study |
| WJ373 | *rph1 ilvG rfb-50* Δ*mepS<>frt mepH-FLAG*<>*frt* | This study |
| WJ374 | *rph1 ilvG rfb-50* Δ*mepM<>frt mepH-FLAG*<>*frt* | This study |

**Plasmid construction**

pHC965 – To construct a template to amplify the 3X FLAG sequence along with a Kan^R^ marker flanked by FRT (flippase recognition target) sites (3X FLAG-aph cassette), the araC-Para sequence of pTB29 was replaced with a synthesized DNA fragment encoding the 3X FLAG sequence. 3X-FLAG DNA was cloned into pTB29 digested with EcoRI and SalI via isothermal assembly.

pWJ20 – The *mepK* gene was amplified using the primer pair 5’- GCTA*TCTAGA*GACCTGTAGACTTGATTATCATG -3’ and 5’- GCTA *AAGCTT*CTACCAGTGCCGTGCTGGCCCGG -3’. The resulting PCR product was digested with XbaI and HindIII and ligated with pHC800 digested with the same enzymes to generate pWJ20. The recognition sequences of the restriction enzymes are underlined and italicized.

pWJ24 – The *mepH* gene was amplified using the primer pair 5’- GCTA*GGATCC*AAGCAAGCCAGGGAGAGTTC -3’ and 5’- GCTA *AAGCTT*AGCGAAGTGTTTTTGGGGTC -3’. The resulting PCR product was digested with BamHI and HindIII and ligated with pTD68 digested with the same enzymes to generate pWJ24.

pWJ25 – The *prc* gene was amplified using the primer pair 5’- GAGGCTCACAGAGAACAGATTGGT*GGATCC*GTAGAAGATATCACGCGTGCTG -3’ and 5’- CAGTGGTGGTGGTGGTGGTGCTCGAC*AAGCTT*ACTTGACGGGAGCGGGTTG -3’. The PCR product was cloned into pTD68 digested with BamHI and HindIII via SLIC (sequence and ligation independent cloning) (Jeong et al., 2012).

pWJ26 – To generate a plasmid that expresses His6-SUMO-tagged *nlpI*, the *nlpI* gene was amplified using the primer pair 5’- GCTA *GGATCC*AATACTTCCTGGCGTAAAAGTG -3’ and 5’- GCTA*AAGCTT*ATTGCTGGTCCGATTCTGCCAG -3’. The resulting PCR product was digested with BamHI and HindIII and ligated with pTD68 digested with the same enzymes to generate pWJ26.

pWJ72 – The *nlpI* gene was amplified with the primer pair 5’-GCTA*CATATG*AAGCCTTTTTTGCGCTGG-3’ and 5’-GCTA*AAGCTT*CTATTGCTGGTCCGATTCTG-3’. The resulting PCR product was digested with NdeI and HindIII, and ligated with pWJ90 digested with the same enzymes to generate pWJ72.

pWJ90 – To construct an empty vector plasmid with the *tac* promoter with a Φ 10 RBS, Ptac::Φ10 RBS sequence was synthesized and cloned into pHC859 digested with XbaI and BamHI via isothermal assembly.

pWJ186 – The *prc* gene was amplified with the Φ10 RBS using the primer pair 5’- TGTGAGCGGATAACAATTCCCC*TCTAGA*TTTAAGAAGGAGATATACATATGAACATGTTTTTTAGGCT -3’ and 5’- GATATTATCGTGAGATCGAT*AAGCTT*TTACTTGACGGGAGCGGGTT -3’. The PCR product was cloned into pWJ90 digested with XbaI and HindIII via SLIC.

**Supplementary Table 2. Plasmids used in this study**

| Plasmid | Genotype* | Origin | Source/  Reference |
| --- | --- | --- | --- |
| pKD13 | *bla, aph* cassette flanked by *frt* sequence | R6K | (Datsenko and Wanner, 2000) |
| pCP20 | *cat bla cl857 λP_R_::FLP* | pSC101(*ts*) | (Datsenko and Wanner, 2000) |
| pTB29 | *bla aph* cassette flanked by *frt* sequence *araC* P*araBAD* | R6K | (Bernhardt and Boer, 2003) |
| pTB102 | *cat cI857 λP_R_::intHK022* | pSC101(*ts*) | (Bernhardt and Boer, 2005) |
| pTD68 | P*_T7_*::*his_6_-sumo*, Amp^R^ | ColE1 | (Uehara et al., 2010) |
| pHC800 | *cat* Ptac::empty | ColE1 | (Lai et al., 2017) |
| pHC859 | *attHK022 tetAR* Ptac::*sulA* | R6K | (Lai et al., 2017) |
| pHC965 | *bla 3X-FLAG aph* cassette flanked by *frt* sequence | R6K | This study |
| pTK1 | *cat* Ptac::*mepM* | ColE1 | (Lai et al., 2017) |
| pTK2 | *cat* Ptac::*mepS* | ColE1 | (Lai et al., 2017) |
| pTK3 | *cat* Ptac::*mepH* | ColE1 | (Lai et al., 2017) |
| pTK4 | *cat* Ptac::*mepA* | ColE1 | (Lai et al., 2017) |
| pTKD1 | *cat* Ptac::*dacB* | ColE1 | (Lai et al., 2017) |
| pTKD4 | *cat* Ptac::*pbpG* | ColE1 | (Lai et al., 2017) |
| pTKD5 | *cat* Ptac::*ampH* | ColE1 | (Lai et al., 2017) |
| pWJ20 | *cat* Ptac::*mepK* | ColE1 | This study |
| pWJ24 | P*_T7_*::*his_6_-sumo*-*mepH*, Amp^R^ | ColE1 | This study |
| pWJ25 | P*_T7_*::*his_6_-sumo*-*prc*, Amp^R^ | ColE1 | This study |
| pWJ26 | P*_T7_*::*his_6_-sumo*-*nlpI*, Amp^R^ | ColE1 | This study |
| pWJ72 | *attHK022 tetAR* Ptac::*nlpI* | R6K | This study |
| pWJ90 | *attHK022 tetAR* Ptac::empty | R6K | This study |
| pWJ186 | *attHK022 tetAR* Ptac::*prc* | R6K | This study |

**Supplementary Table 3. PCR primers for recombineering***

| Target Gene | Primer 1 | Primer 2 |
| --- | --- | --- |
| **Primers for gene deletion** | | |
| mepS | TATTTGTCGTTAAGGACTTCAAGGGAAAACAAACAACATGATTCCGGGGATCCGTCGACC | ATTGCATCCAAACGGTTTATTAGCTGCGGCTGAGAACCCGTGTAGGCTGGAGCTGCTTC |
| mepM | GAGCTGCCTGAAAGGAGATTAATGAGGAAGTGATTACGTGATTCCGGGGATCCGTCGACC | GGCTGCGAATGGATGTTAATTAATCAAACCGTAGCTGCGGTGTAGGCTGGAGCTGCTTC |
| prc | GAACACCTGGTGTTCTGAAACGGAGGCCGGGCCAGGCATGATTCCGGGGATCCGTCGACC | TTCTTGTGCCTGATTGATATTACTTGACGGGAGCGGGTTGTGTGTAGGCTGGAGCTGCTT |
| nlpI | AGGACGTTCATTCAACCGTGGTCTTCGGGAGTGGGAAATGATTCCGGGGATCCGTCGACC | GGGCTGATGTGTACGTCAGCTATTGCTGGTCCGATTCTGCTGTAGGCTGGAGCTGCTTC |
| dacB | GTATGACGGCTCGATTCCAGGTTGTTAGCGCGAGATTATGATTCCGGGGATCCGTCGACC | TGCCGGGGTTTCTTTTTGACTAATTGTTCTGATAAATATCTGTAGGCTGGAGCTGCTTC |
| pbpG | CAACCCGTGCGCGTGAACCACTATCTGAATGCTCATCATGATTCCGGGGATCCGTCGACC | TATCGCCATCCGAATTCACTTAATCGTTCTGTGCCGTCTGTGTAGGCTGGAGCTGCTTC |
| mepA | TGCCGATGTGAAGACTGATATTCCACGCTGGTAAAAAATGATTCCGGGGATCCGTCGACC | AGGCTATTAAACGTTTCCATTAGATCACGTGCTCATCCAGTGTAGGCTGGAGCTGCTTC |
| ampH | CGCTCAGTTACCTTCATTCAATCTATGGACACCACCGTTGATTCCGGGGATCCGTCGACC | TTTGCCTTCAGAGTATTAATCAGGACGCGGGGATAACCAATGTAGGCTGGAGCTGCTTC |
| mepH | CAGGCATGATAGACCTGCCTTTACAGAGGGACGCTCAGTGATTCCGGGGATCCGTCGACC | GTAACAACAGGGTAAAGTTTTAGCGAAGTGTTTTTGGGGTGTAGGCTGGAGCTGCTTC |
| mepK | ATATAAACGATAACATTGACCTGTAGACTTGATTATCATGATTCCGGGGATCCGTCGACC | CTGTTTCGTTAAGCAATTACTACCAGTGCCGTGCTGGCCCTGTAGGCTGGAGCTGCTTC |
| **Primers for replacing the native promoter with an arabinose-inducible promoter** | | |
| mepS | CACAGTAATTAACATCATCAGGGTTATTTTTATAGTGAGGGTGTAGGCTGGAGCTGCTTC | ATCGGTTGAGATTTGACCATGTTGTTTGTTTTCCCTTGAAATCGCCGAATTCGCTAGCCC |
| **Primers for 3X-FLAG tagging** | | |
| mepH | TGTTGGCGCTCGTCGGGTAATGACCCCAAAAACACTTCGCGACTACAAAGACCATGACGG | AGATGAACTTACCCTGTTGCCGTAACAACAGGGTAAAGTTGTGTAGGCTGGAGCTGCTTC |
| pbpG | CGCCCAAATGGCGGCGGCGGGGCAGACGGCACAGAACGATGACTACAAAGACCATGACGG | TAAAAATTACGGATGGCAGAGTATCGCCATCCGAATTCACGTGTAGGCTGGAGCTGCTTC |
| mepA | GCCGCCTTCCTGCCAGGCGCTACTGGATGAGCACGTGATCGACTACAAAGACCATGACGG | ACAGCGGGGAAACCATAAACAGGCTATTAAACGTTTCCATGTGTAGGCTGGAGCTGCTTC |
| ampH | CGAGCTAAGCGGGAATAAACCGTTGGTTATCCCCGCGTCCGACTACAAAGACCATGACGG | CAAACTGGTCGCTATCAACCGTTTGCCTTCAGAGTATTAAGTGTAGGCTGGAGCTGCTTC |
| dacB | TTTTGAAAGCCGTTTGTATAAAGATATTTATCAGAACAATGACTACAAAGACCATGACGG | TAATCTGAAGCCCCGGCCATGTGCCGGGGTTTCTTTTTGAGTGTAGGCTGGAGCTGCTTC |

* Underlined are sequences homologous to chromosomal DNA.

**REFERENCES**

Bernhardt, T. G., and Boer, P. A. J. de (2005). SlmA, a Nucleoid-Associated, FtsZ Binding Protein Required for Blocking Septal Ring Assembly over Chromosomes in E. coli. *Mol Cell* 18, 555–564. doi:10.1016/j.molcel.2005.04.012.

Bernhardt, T. G., and Boer, P. A. J. D. (2003). The Escherichia coli amidase AmiC is a periplasmic septal ring component exported via the twin‐arginine transport pathway. *Mol Microbiol* 48, 1171–1182. doi:10.1046/j.1365-2958.2003.03511.x.

Bernhardt, T. G., and Boer, P. A. J. D. (2004). Screening for synthetic lethal mutants in Escherichia coli and identification of EnvC (YibP) as a periplasmic septal ring factor with murein hydrolase activity. *Mol Microbiol* 52, 1255–1269. doi:10.1111/j.1365-2958.2004.04063.x.

Datsenko, K. A., and Wanner, B. L. (2000). One-step inactivation of chromosomal genes in Escherichia coli K-12 using PCR products. *Proc National Acad Sci* 97, 6640–6645. doi:10.1073/pnas.120163297.

Ferrières, L., Hémery, G., Nham, T., Guérout, A.-M., Mazel, D., Beloin, C., et al. (2010). Silent Mischief: Bacteriophage Mu Insertions Contaminate Products of Escherichia coli Random Mutagenesis Performed Using Suicidal Transposon Delivery Plasmids Mobilized by Broad-Host-Range RP4 Conjugative Machinery. *J Bacteriol* 192, 6418–6427. doi:10.1128/jb.00621-10.

Guyer, M. S., Reed, R. R., Steitz, J. A., and Low, K. B. (1981). Identification of a Sex-factor-affinity Site in E. coli as   . *Cold Spring Harb Sym* 45, 135–140. doi:10.1101/sqb.1981.045.01.022.

Jeong, J.-Y., Yim, H.-S., Ryu, J.-Y., Lee, H. S., Lee, J.-H., Seen, D.-S., et al. (2012). One-Step Sequence- and Ligation-Independent Cloning as a Rapid and Versatile Cloning Method for Functional Genomics Studies. *Appl Environ Microb* 78, 5440–5443. doi:10.1128/aem.00844-12.

Johnson, J. E., Lackner, L. L., Hale, C. A., and Boer, P. A. J. de (2004). ZipA Is Required for Targeting of D MinC/DicB, but Not D MinC/MinD, Complexes to Septal Ring Assemblies in Escherichia coli. *J Bacteriol* 186, 2418–2429. doi:10.1128/jb.186.8.2418-2429.2004.

Lai, G. C., Cho, H., and Bernhardt, T. G. (2017). The mecillinam resistome reveals a role for peptidoglycan endopeptidases in stimulating cell wall synthesis in Escherichia coli. *Plos Genet* 13, e1006934. doi:10.1371/journal.pgen.1006934.

Pal, D., Venkova-Canova, T., Srivastava, P., and Chattoraj, D. K. (2005). Multipartite Regulation of rctB, the Replication Initiator Gene of Vibrio cholerae Chromosome II. *J Bacteriol* 187, 7167–7175. doi:10.1128/jb.187.21.7167-7175.2005.

Uehara, T., Parzych, K. R., Dinh, T., and Bernhardt, T. G. (2010). Daughter cell separation is controlled by cytokinetic ring‐activated cell wall hydrolysis. *Embo J* 29, 1412–1422. doi:10.1038/emboj.2010.36.
